# Oscillatory activity, phase differences, and phase resetting in the inferior olivary nucleus

**DOI:** 10.3389/fnsys.2013.00022

**Published:** 2013-06-19

**Authors:** Yaara Lefler, Benjamin Torben-Nielsen, Yosef Yarom

**Affiliations:** ^1^Department of Neurobiology, The Institute of Life Sciences, The Edmond and Lily Safra Center for Brain Sciences, The Hebrew UniversityJerusalem, Israel; ^2^Blue Brain Project, Brain Mind Institute, École Polytechnique Fédérale de LausanneLausanne, Switzerland

**Keywords:** sub-threshold oscillation, inferior olive, cerebellum, phase resetting, timing

## Abstract

The generation of temporal patterns is one of the most fascinating functions of the brain. Unlike the response to external stimuli temporal patterns are generated within the system and recalled for a specific use. To generate temporal patterns one needs a timing machine, a “master clock” that determines the temporal framework within which temporal patterns can be generated and implemented. Here we present the concept that in this putative “master clock” phase and frequency interact to generate temporal patterns. We define the requirements for a neuronal “master clock” to be both reliable and versatile. We introduce this concept within the inferior olive nucleus which at least by some scientists is regarded as the source of timing for cerebellar function. We review the basic properties of the subthreshold oscillation recorded from olivary neurons, analyze the phase relationships between neurons and demonstrate that the phase and onset of oscillation is tightly controlled by synaptic input. These properties endowed the olivary nucleus with the ability to act as a “master clock.”

## Introduction

Several theories are concurrently being used to explain how neuronal communication is organized and how information is coded in the brain. Regardless the differences between those theories, one consensus is that timing *per se* is a hallmark of brain function: whatever the brain is doing—learning, executing motor commands, or retrieving memory—the signals have to be processed at the right place and at the right time. In focusing on timing, we can distinguish a “timely response” from a generation of “temporal patterns.” The former is a reflexive response while the latter is manifested in the typical intrinsically generated rhythmic activity observed in various brain structures.

Intrinsic rhythmic activity, which is generated by complex interactions between cellular properties and network dynamics, can serve as a “master clock” capable of generating temporal patterns. We identify three functional requirements for a master clock. First, the clock must maintain an accurate and continuous carrier frequency that acts as a reference signal. Second, the temporal precision of a signal cannot be higher than the clock's period. However, if the clock is implemented by multiple units, and a stable phase-offset is maintained between those units, a higher temporal precision can be achieved. Third, in order to correlate the timing signals with a specific operation of the system, the onset and termination of the clock has to be controlled.

In this manuscript we make the case that such a master clock can reside in the inferior olive nucleus. Following background information on olivary activity, we discuss the first two requirements by reviewing previous work. Finally, the third requirement is discussed in the light of new experimental observations. We complement these observations with network simulations of the inferior olive and highlight its capability to function as a master clock in the brain.

## Background information

**Figure 1:**
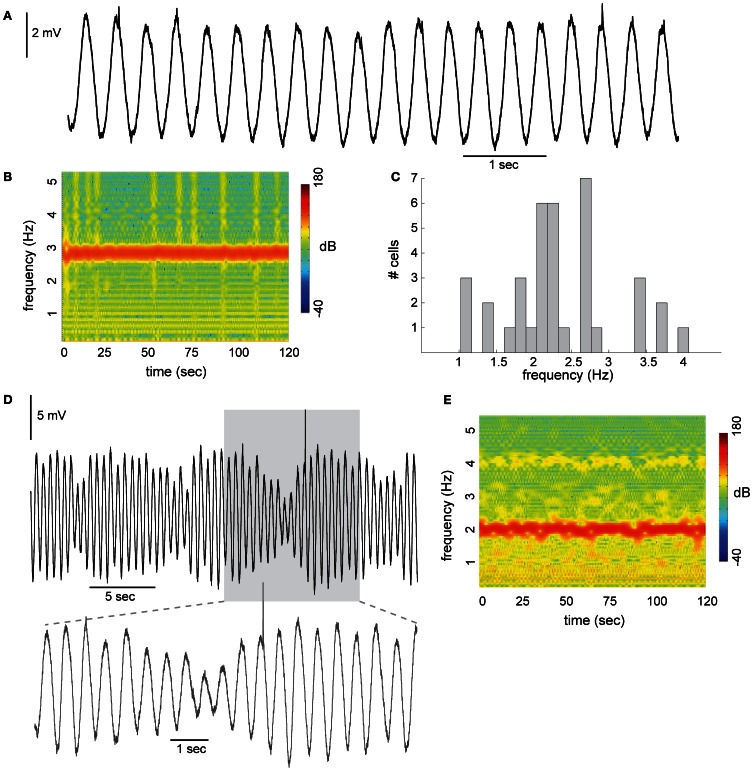
**Subthreshold oscillations (STO) in neurons of the inferior olive. (A)** Stable sinusoidal like oscillations with constant amplitude recorded from olivary neuron in slice preparation. **(B)** Spectrogram of the frequency component during 120 s of recording. **(C)** Distribution of STO frequency recorded from 37 olivary neurons. Average frequency was 2.4 Hz (histogram bin size is 1/6 Hz). **(D)** STO with amplitude modulation. Prolonged recording, showing the rhythmicity of the amplitude modulation. Lower panel, the gray area in **(D)**, displayed at faster time scale. **(E)** Spectrogram of the example shown in **(D)**.

It is often suggested that the abilities to measure time or to generate signals at an appropriate time, are manifestations of cerebellar computational processes (Ivry and Keele, 1989; Mauk et al., 2000). The result of this computation is a train of impulses that has a specific temporal pattern tailored to the task at hand. There is, however, an ongoing debate about the neural mechanism that subserves this timing function. One possibility is that the parallel fibers act as delay lines, sequentially activating different Purkinje cells at accurate time intervals (Braitenberg, 1967). An alternative possibility is the use of the coupled oscillators of the olivary nucleus to generate temporal patterns (Jacobson et al., 2008; Llinas, 2011). The uniqueness of this nucleus lies in the richness of the interacting membrane currents combined with electrical synapses between olivary cells, which can generate a large repertoire of electrical behaviors. One of these fascinating electrical behaviors is the subthreshold membrane potential oscillation (STO). The STO in the inferior olive is a precise, almost perfect sinusoidal alteration in the membrane potential and is unprecedented in neuronal electrical activity (Llinas and Yarom, 1986; Chorev et al., 2007; Khosrovani et al., 2007 and see Figure 1A). Although the amplitude of the oscillation varies, the frequency itself remains stable (Benardo and Foster, 1986; Placantonakis et al., 2006; Khosrovani et al., 2007 and see below). It is of no wonder that the olivary STO has been associated with timing function accredited to the cerebellum (Welsh et al., 1995; Yarom and Cohen, 2002; Jacobson et al., 2008; Hansel, 2009; Llinas, 2009).

Different strategies are proposed in which the inferior olive STO relates to precise timing in the cerebellar system. In earlier work, we demonstrated that in individual neurons, the synaptic potentials integrate with voltage oscillation in such a way that the output was phase-locked to the oscillation while being independent of the actual input phase (Lampl and Yarom, 1993). In another work, it has been proposed that the number of spikes in a burst of activity that ascends through the climbing fiber reflects the phase of the oscillation (Mathy et al., 2009). Moreover, the number of spikes in a burst can control the extent and polarity of climbing-fiber induced plasticity (but see Bazzigaluppi et al., 2012).

**Figure 2:**
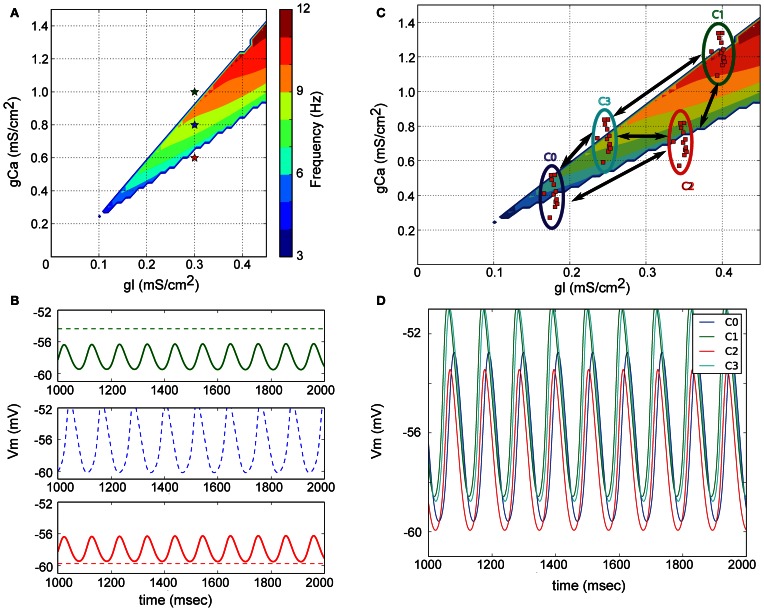
**Origin of subthreshold membrane potential oscillations in a model of the inferior olive. (A)** Model neurons only contain a leak and low-threshold calcium current. The parameter space for the associated conductances is illustrated. The colored region delineates the region in parameter space where neurons spontaneously oscillate. **(B)** Non-oscillating neurons can become oscillators due to electrical coupling. Color of the membrane potential traces correspond to the color of the indicated points in **(A)**. Dashed line is the potential of the cell when not coupled; full line after coupling. The model neurons indicated with red and green in **(A)** only oscillate when electrically coupled. The frequency of the emerging oscillation depends on the exact coupling strength. **(C)** Network model of the IO. Neurons with similar electrical profiles (=similar conductance densities) form clusters and are abundantly interconnected inside each cluster. Sparser connections are added between neurons belonging to distinct clusters. **(D)** Membrane potential of one neuron from each cluster. Network wide coherent oscillations emerge due to the intricate electrical coupling between neurons and clusters. Neurons belonging to different cluster maintain a stable phase difference between them [Reproduced from Torben-Nielsen et al. (2012)].

The neuronal mechanism underlying STOs is also debated. The disagreement is whether all olivary neurons oscillate and act as individual clocks, or whether the STO is an emergent property of the network. Both possibilities are supported by experimental (Bleasel and Pettigrew, 1992; Lampl and Yarom, 1997; Long et al., 2002; De Zeeuw et al., 2003; Leznik and Llinas, 2005; Placantonakis et al., 2006; Marshall et al., 2007), and modeling work (Yarom, 1991; Manor et al., 1997; Makarenko and Llinas, 1998; Schweighofer et al., 1999; Loewenstein et al., 2001). We theoretically predicted (see also Figure 2) and experimentally confirmed (Chorev et al., 2006) that electrical coupling between olivary cells could promote the generation of STO.

Regardless of the strategy of how to convey the timing signal, or on how the timing signal is established, the accuracy and repeatability of the oscillations strongly remind us of clock like activity. A potential pitfall for a clock based on STO is that the temporal precision is limited by the period of the clock: the precision cannot exceed the period. For instance, an oscillator with a frequency of 10 Hz cannot reach a temporal precision shorter than 100 ms. In case the master clock is realized as collective rather than as individual entities, a group of oscillators with similar frequency operating at different phases can increase the somewhat limited temporal resolution. Indeed, by having multiple oscillators with the same period and offset in between of their phase, the whole period can be spanned by such a collection of oscillations.

In the following sections we will demonstrate that the olivary nucleus fulfills the needed criteria to operate as a “master clock” for the cerebellar system.

### Requirement 1: stable carrier frequency

A typical example of STO with an almost sinusoidal waveform recorded from inferior olivary neuron is shown in Figure 1A. The frequency in this example was 2.8 Hz and the amplitude varies from 4.3 to 5.1 mV. STO were encountered in 39% (75/194) of the recorded neurons. The spectrogram shown in Figure 1B demonstrates the remarkable stability of the STO. During the two minutes of recording the average frequency was 2.8 Hz and varied slightly within a narrow range of 2.6–3.0 Hz. On a population level (Figure 1C; *n* = 37) the average frequency is distributed normally over a rather wide range of 1–4 Hz (average is 2.4 Hz; measured in slice preparation at room temperature). In contrast to the stability of the frequency of oscillations, amplitude modulations were frequently observed (Figure 1D). The amplitude modulation often appeared in a form of slow rhythmic changes, resembling beating oscillations. In this example the amplitude modulation followed a frequency of about 0.15 Hz where the amplitude varies from 2.3 to 7.5 mV. Yet, the stability of the frequency was maintained (Figure 1E). It is worth noting that the frequency of the slow modulation is within the frequency differences between individual neurons (Figure 1C), suggesting that beating oscillations may reflect the behavior of coupled oscillators.

Building on the previous work (Manor et al., 1997) we also developed a network model of the inferior olive with which we investigated the generation of STO (Torben-Nielsen et al., 2012). The concept of this model is schematically described in Figure 2A. Within the parameter space of leak conductance and calcium conductance (low-threshold T-type), one can define four types of neuronal behavior (stable, conditional bistable, conditional oscillator, and spontaneous oscillator Manor et al., 1997) of which only one, located within the colored area, displays spontaneous oscillations. An example of the behavior of three neurons (red, blue, and green stars in Figure 2A) from three different areas is shown in Figure 2B (dashed lines) where only the blue marked neuron oscillates without coupling. When the non-oscillating model neurons (red and green stars) are electrically coupled, they oscillate at the same frequency and roughly the same phase. The emergent network oscillations will occur as long as the “average” electrical profile (weighted by the strength of the electrical coupling) lies in the region of the parameter space where a neuron oscillates spontaneously (which in Figure 2A is close to the location of the blue star). Hence, IO neurons can be spontaneous oscillators or not; the network oscillation emerges from the electrical coupling. The resultant oscillation has a stable frequency over time and can thus act as a reference time signal.

### Requirement 2: stable phase differences

**Figure 3:**
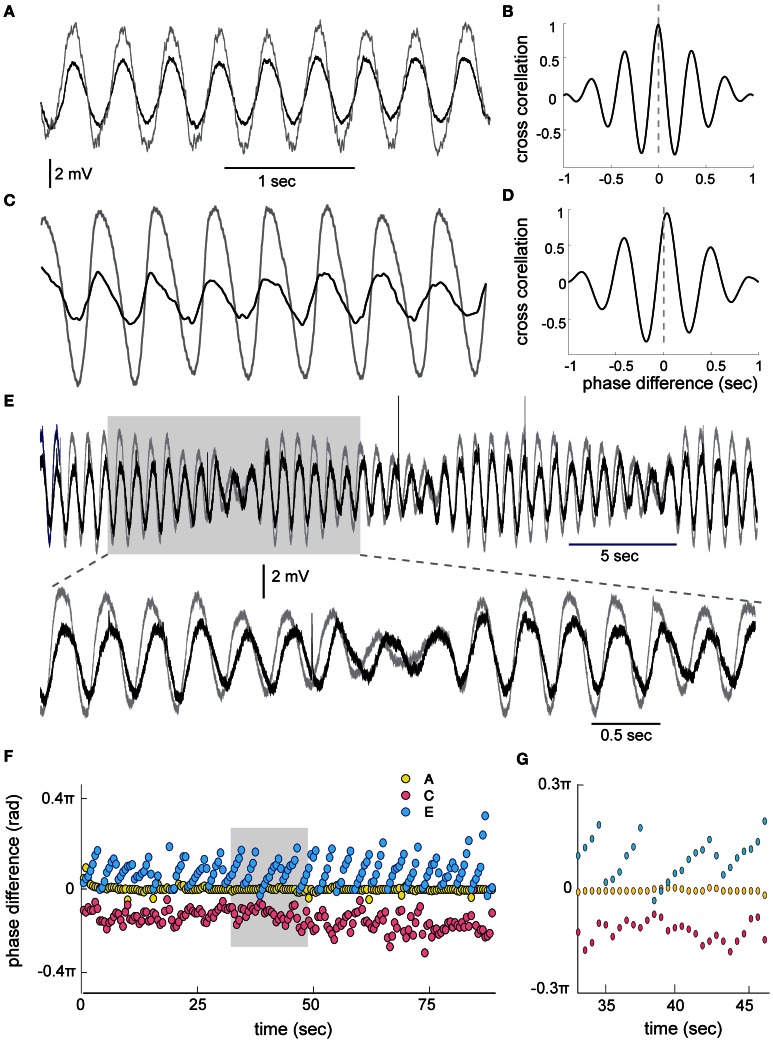
**Phase relationship between STO in 2 simultaneously recorded olivary cells. (A)** An example of zero phase difference between two cells. **(B)** The cross correlation calculated between the two waves on 1 s of recording showing the zero phase. **(C,D)** The same as in **(A,B)** for two cells showing approximately 0.07 × 2π (33 ms) phase difference (STD ± 0.02). **(E)** An example of STO in two cells displaying slow amplitude modulation. The gray area in **(E)** is displayed at faster time scale in the lower trace. Note the progressive increase in phase during the cycle of amplitude modulation. **(F)** Phase difference, calculated using cross correlations of 1 s in 50% overlap, throughout 85 s of recording for the three examples shown in A (yellow circles), C (red circles), and E (blue circles). **(G)** The gray area in **(F)** displayed at faster time scale.

A simultaneous recording from two oscillating neurons (Figures 3A,C,E) shows that they oscillate at the same frequency. We subdivided the pairs of oscillating cells into two groups: the stable amplitude and the modulated amplitude. The case of stable amplitude and zero phase difference (Figures 3A,B,F, yellow circles) exhibited high stability throughout the recording session (0.001 ± 0.007 × 2π). On the other hand, a reduced stability was observed in the case of non-zero phase difference (Figures 3C,D,F, red circles, 0.07 ± 0.02 × 2π). The latter is in agreement with our previous publication (Jacobson et al., 2009). In the cases of modulated amplitude, the phase followed closely the slow rhythm of the amplitude modulation (Figure 3F, blue circles). At the point in which the minimal amplitude of the two waves coincides, the phase was equal. As the amplitude increases, the phase difference increases up to the point of the next minimal amplitude where it immediately drops back to zero phase. This rhythmic alteration in phase persists throughout 85 s of recordings (Figures 3F,G).

The phase difference between oscillating neurons can be reproduced in our simplified model of olivary network, provided that the network model represents anatomical and physiological data about the IO. The anatomical data dictated a clustered network (Sotelo et al., 1974), whereas the physiological data suggests that neurons within a cluster have similar electrical profiles (Manor et al., 2000; Devor and Yarom, 2002) and are abundantly connected to each other, while only sparsely connected to neurons in other clusters (Figure 2C). As shown in Figure 2D this extended model can generate STO where neurons oscillate at the same frequency but with different phase. The phase difference results from the difference in calcium-conductance: neurons with higher density depolarize quicker than neurons with a lower calcium-conductance density. As a consequence, the neurons with the high calcium-conductance density are advanced in their phase. Thus, this simplified neuronal model can reconstruct two main observations: oscillation and phase, providing a mechanistic explanation for both.

### Requirement 3: control over oscillation by reset

**Figure 4:**
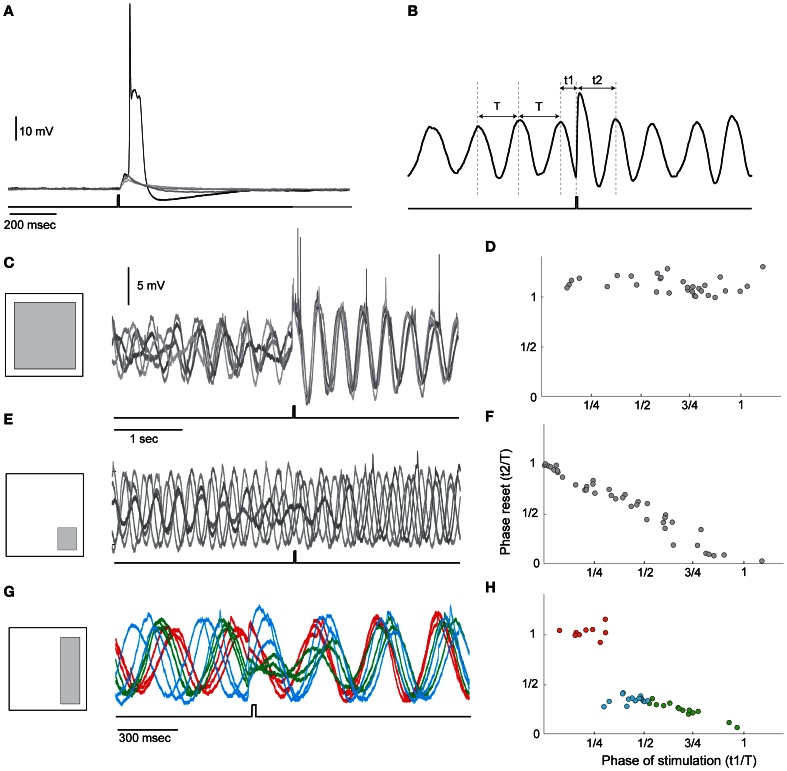
**Phase resetting of the STO by light stimulation. (A)** Superimposed traces of olivary neuron responses to a single pulse of light (lower trace). Led blue light (460 nm) was delivered to the slice using Digital Mirror Light Stimulator to illuminate specific areas at the resolution of 20 × 20 micron in ×40 objective. The system enables simultaneous illumination of multiple areas at a time resolution of 2 ms. Note that the synaptic potential occasionally reached the threshold for spike initiation. **(B)** The method used to quantify the resetting of the STO by light. T is the cycle period and t1 and t2 are the time of the stimulus and the time of the first peak after the stimulus, respectively. **(C)** Left, Schematic illustration of the illuminated area; right, superimposed traces showing the resetting effect of the pulse of light. Since the stimulus frequency is uncorrelated with the frequency of the STO, each stimulus appears at different phase of the STO. The complete overlap of the traces after the stimulus indicates that the stimulus reset the STO. **(D)** Plotting t2 as a function of t1 demonstrates that t2 is equal to 1 irrespectively of the time of stimulation. **(E,F)** and **(G,H)** are the same as **(C,D)** for different illuminated areas. In **(G)** the traces are color coded according to the stimulation phase: where red is used when stimulation occurred at the first quarter of the cycle and a complete resetting was induced; green is used for the second quarter of cycle and blue for the ascending half of the period.

This essential requirement stems from the need to correlate the timing signals with a specific operation of the system. Realizing this ability in neuronal systems means that synaptic inputs should be able to modulate the oscillatory activity. We examined this possibility in an *in vitro* system using optogenetic approach. We found that olivary neurons in Thy1-COP4/EYFP transgenic mice that express ChR2 in various brain structures are unlabeled. This favorable condition implies that synaptic input into the olivary neurons can be evoked by light stimulation without the direct activation of the neurons. Indeed, as shown in Figure 4A, a light pulse delivered to a restricted area of the olivary nucleus reliably evoked a synaptic response that can occasionally reach threshold and trigger an action potential. We used this system to study the interaction between the oscillatory activity and synaptic inputs. The five superimposed traces in Figure 4C show that in response to a single pulse of light (lower trace) a phase resetting occurred. The method we used to quantify the resetting effect is shown in Figure 4B. We measured the interval between the time of the stimulus and the last peak of the oscillation (t1) and the time from the stimulus to the next peak of oscillation (t2). After normalizing these two parameters (t1 and t2) by the period of the oscillations (T) we plotted t2 as a function of t1. In an effective reset mechanism, t2 is equal to 1, independent of t1. If no reset occurs, t2 is negatively related to t1 with a slope of −1. In the example of Figures 4C,D, t2 is independent of t1 and as shown in the superimposed traces the oscillatory activity after the stimulus is completely overlaps while before the stimulus it is uncorrelated. A different example is shown in Figures 4E,F in which the pulse of light failed to induce resetting and the negative linear relation was obtained.

The ability of synaptic input to reset the phase of oscillation depends on the synaptic pathway that is activated by the stimulus. Different synaptic pathways can be activated by redirecting the light pulse to different areas in the visual field. In fact, the different behavior shown in Figure 4 was generated by shifting the light to different area as indicated in the left panels in **(C**,**E,G)**; Whereas an efficient resetting was obtained when a large area was illuminated (Figures 4C,D), it was totally ineffective when the lower right corner of the visual field was illuminated (Figures 4E,F). As shown in Figures 4G,H, a step like function was measured when the light was directed to the right side of the visual field. These findings suggest that either a specific input provides the resetting ability or that the number of activated cells determined the resetting efficiency. The latter possibility implies that it is the network dynamic that generates the required activity rather than the activity of individual neurons. As mentioned above in this specific experimental system the light pulse activates several different types of synapses, including excitatory, inhibitory and neuromodulatory pathways. We found that while blocking GABA receptors has little or no effect, blocking Glutamate receptors with CNQX completely abolished the ability of the light pulse to reset the oscillations.

**Figure 5:**
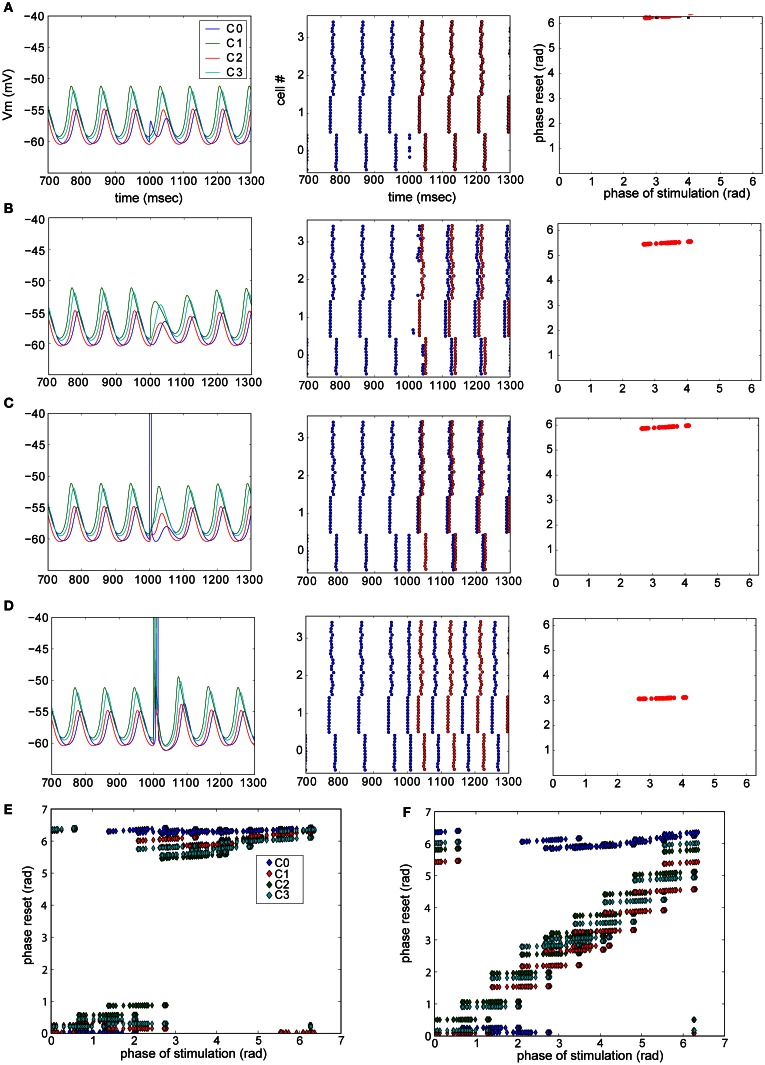
**Phase resetting in a model of the olivary nucleus.** Phase of the network reset by strength of excitation and post-synaptic cell profile. **(A–D)** Examples of resets in the network model. Left column: membrane potential of one neuron of each cluster. Center panel: raster plot showing the peaks of the STO. Blue dots are the actual peaks, while red dots represent the predicted peaks given there was no network reset. Right panel: summarized phase shift of the STO caused by the reset illustrated in the left and center panel. A weak excitatory pulse was provided to each cell in cluster 0 **(A)** or 1 **(B)**, while a strong, spike-triggering stimulus was presented to cluster 0 **(C)** and 1 **(D)**. **(E,F)** Summary plots of triggered phase-shifts after stimulating each cell in each cluster (one cluster at a time, clusters are color coded) weakly **(E)** or strongly **(F)** [Reproduced from Torben-Nielsen et al. (2012)].

In the experiments, three cases where observed: no reset (Figure 4F), phase-independent reset (Figure 4D), and phase-dependent reset (Figure 4H). We further examined the detailed resetting mechanism by using our simplified model of the olivary nucleus. For instance, in the case of phase-independent reset (Figure 4D), what is the new phase compared to the old phase before the reset? To this end we added excitatory synapses to all neurons in one cluster at a time and observed the phase-shift in the oscillation after evoking a weak (not triggering a spike) or a strong (triggering a spike) synaptic event (Figure 5). We found that depending on which cluster received the synaptic event, the network could be reset, even with a weak pulse. Also, the difference between the old (before excitation) and new phase depend on the exact timing of the excitation (Figures 5E,F). As shown in Figure 5F, the reset could cause the largest possible phase-shift (i.e., the new phase is in complete anti-phase with the phase before the excitation). Thus, we can conclude that excitation can reset the network, and, that the magnitude of the reset depends on the strength and timing of the excitatory inputs.

## Concluding remarks

We argue that the inferior olive operates as a “master clock,” providing timed signals needed by the cerebellum to perform its role in temporal coordination of brain and body functions. Two main features contribute to the temporal precision of the “master clock”: The accuracy and stability of the carrier frequency and the phase relationships between the constituents of the “master clock.” As we hold the notion that the oscillatory activity reflects network architecture and dynamics, we claim that this network arrangement is the main contributor to the stability of the carrier frequency. Neurons are noisy elements and if we seek for uncompromised accuracy we cannot depend on the capricious nature of single neurons. In a way, having a network based oscillation, the inferior olive averages out the noise of single neuron to establish a reliable rhythm. It can be argued that averaging might also eliminate phase differences, however, if we adopt the concept of clustered organization where neurons within a cluster have similar properties and are mutually connected to each other (see Figure 2C), we can gain stability within the cluster and maintain phase differences between clusters.

As mentioned throughout this manuscript, the phase difference plays a pivotal role in the ability of the “master clock” to provide time intervals shorter than the cycle duration. In some occasions the phase difference is remarkably stable. In others, particularly when phase differences exist, random variability was observed. Despite this variability, phase differences increase the time resolution of the clock. In the example shown in Figure 3F, the oscillation period of 370 ms was associated with a phase difference of 33 ± 5 ms. These numbers entail an order of magnitude increase in the clock's precision. The rhythmic changes in phase occurred exclusively in those cases where rhythmic modulation in the amplitude was observed. We suggest that this pattern of beating oscillations can be explained by coupling of two oscillators that differ in their carrier frequency. However, coupled oscillators cannot, in a simple way, account for the rhythmic phase changes. Regardless of the mechanism of rhythmic changes in phase, and assuming that it is not a peculiarity of the *in vitro* system, it does endow the system with a whole new dimension of temporal abilities. A device that can automatically generate patterned activity with systematic increase in inter-event intervals is of great importance. For example, it can provide a tool for accelerating or decelerating (motor) command types.

Using optogenetic approach we confirmed resetting of STO by synaptic input (Kazantsev et al., 2004; Khosrovani et al., 2007). There are two clear take-home messages here. The phase of oscillations can be reset by excitatory synaptic input and the resetting, at least partially, depends on the phase of the stimulus. In addition to the need to time the temporal patterns with the task in hand, it also enables it to fine tune the pattern by adjusting the phase in correlation to the ongoing activity. The ability of the system to re-adjust the phase and closely control the frequency, although within a narrow range, endows the timing device with the ability to operate as accurate, yet modifiable, timing machine.

It should be noted that the observations described here are based on *in vitro* study. To what extent does the remarkable STO behavior remain intact in the *in vivo* situation? In an earlier study we described phase differences in complex spike activity recorded from the cerebellar Purkinje cells in freely moving animals. Although it was under harmaline intoxication, it did show phase differences in complex spike activity and demonstrated that the remarkable stability of the phase (Jacobson et al., 2009). Since the Purkinje cells complex spike is the manifestation of olivary activity in the cerebellar cortex, it suggests that the STO and their phase differences are reliably transmitted at least across the first synapse downstream to the olivary nucleus. Therefore, the thorough understanding of the STO, their frequency and phase stability as well as the mechanisms of resetting will pave the way for understanding the cerebellar role in timing of brain functions.

### Conflict of interest statement

The authors declare that the research was conducted in the absence of any commercial or financial relationships that could be construed as a potential conflict of interest.
